# Beliefs, barriers, and behaviors: Exploring dementia prevention perspectives in culturally diverse populations in The Netherlands

**DOI:** 10.1177/13872877261443983

**Published:** 2026-04-23

**Authors:** Najoua Lazaar, Awaale F. Rirash, Janne M. Papma, Francesco U. S. Mattace Raso, Sanne Franzen

**Affiliations:** 1 Department of Neurology and Alzheimer Center, 6993 Erasmus MC University Medical Center, Rotterdam, the Netherlands; 2 Department of Geriatrics and Alzheimer Center, 6993 Erasmus MC University Medical Center, Rotterdam, the Netherlands

**Keywords:** Alzheimer's disease, cultural diversity, dementia, healthy aging, lifestyle, primary prevention

## Abstract

**Background:**

Modifiable risk factors play a key role in preventing or delaying dementia, yet little is known about how culturally diverse populations in Europe perceive these risk factors.

**Objective:**

This study aimed to: (1) explore lay perceptions of dementia and its risk factors, and (2) examine how these perceptions interact with social and structural determinants of health to shape dementia prevention behaviors.

**Methods:**

We conducted a qualitative study guided by the I-Change model, using semi-structured interviews with 20 adults varying in cultural and linguistic background, education, and age. Interviews were conducted in Dutch, English, and Moroccan-Arabic (*Darija*), translated if needed and transcribed verbatim. Data analysis followed open, axial, and selective coding to identify themes.

**Results:**

Participants emphasized genetics and biological vulnerability over modifiable risk factors when discussing dementia risk. Socioeconomic status, physical ability, and (social) environment were perceived to shape opportunities for healthy aging. Peers, family, and cultural norms were both facilitators and barriers to health-promoting behaviors. Lived- and observed experiences of illness motivated general behavior change, not specifically linked to dementia and Alzheimer's disease. Stress, sleep, resilience, and discipline were viewed as influential for cognitive health. Participants often integrated personal, cultural, and social perspectives when interpreting dementia risk.

**Conclusions:**

Perceptions of dementia and Alzheimer's disease risk among culturally diverse individuals are shaped by biological beliefs, psychosocial influences, and social context. Knowledge of dementia and modifiable risk factors remains limited among minoritized populations. Public health initiatives might benefit from incorporating prevention messages in a community perspective to enhance engagement and motivation.

## Introduction

Modifiable risk factors play a crucial role in preventing or delaying the onset and progression of dementia.^1,2^ These factors include hypertension, obesity, diabetes, high cholesterol, smoking, excessive alcohol consumption, an unhealthy diet, physical inactivity, limited social contact, and minimal cognitive stimulation.^3,4^ Evidence on additional modifiable risk factors, such as air pollution, hearing impairment, traumatic brain injury, and depression, has also emerged.^1,2^ Lifestyle modifications may be particularly impactful in those most at risk of developing dementia, including many minoritized populations globally, such as Latino and African American/Black individuals in the US,^5–7^ migrants in Europe,^8^ and Aboriginal Australians and Torres Strait Islanders in Australia.^9,10^

The motivation to adopt and sustain lifestyle changes is shaped by individuals’ perceptions and understanding of dementia, which are in turn influenced by sociocultural factors such as level of education, gender, religion, and culturally embedded norms.^11^ Different explanatory models emerge from such perceptions, shaping how individuals understand and interpret dementia as a health condition or disease. For example, a study among culturally diverse dementia caregivers in the Netherlands demonstrated that some believed dementia to be caused by medication use, genetic predisposition, or physical problems such as dehydration or sexually transmitted diseases,^12,13^ while others perceived dementia as stemming from an interplay between life events, personality traits, and social and psychological factors. Other notable examples included spiritual explanations of dementia and dementia as “*normal aging”*.^13^ Taking the latter as an example, if someone believes that dementia is just a part of “*normal aging”* and if that person is unaware of the potential benefits of lifestyle changes, they may be unlikely to adopt preventive strategies.^14^ Understanding how illness perceptions influence health behaviors is therefore important.

The I-Change Model provides a useful framework for understanding health behavior change, as it integrates the role of awareness, motivation, and action, including determinants such as illness perceptions and dementia literacy—the knowledge and beliefs that support recognition, management and prevention of dementia.^15^ In the pre-motivational (awareness) phase, people first become aware of health risks and their own behaviors. Risk perceptions interact with knowledge, awareness of one's own behavior, and perceived cues before motivation to change is formed. Dementia literacy (a disease-specific aspect of health literacy), particularly cognitive and emotional representations of dementia held by individuals,^16,17^ may play an important role in this phase. Intentions are subsequently translated into preventive behaviors during the action phase. The success of this translation depends on personal, social and environmental facilitators and barriers.

Behavior change should always be evaluated in context as minoritized individuals may face barriers, such as limited safe spaces for exercise, restricted access to health care, and lack of affordable healthy foods, that can hinder the translation of dementia literacy into effective preventive actions.^18^ Although the scientific community recognizes the importance of these factors,^19^ little is known about which social and structural factors individuals themselves perceive as major barriers to adopting lifestyle changes. Furthermore, more knowledge is needed considering the understanding of dementia and its risk factors in diverse populations.

The aims of this study were therefore: 1) to explore lay perceptions of dementia and its risk factors in culturally diverse populations, and 2) examine how lay narratives about the causes of dementia feature social and structural determinants of health (SSDOH) as factors shaping prevention possibilities.

## Methods

The data for this study were collected through semi-structured, in-depth interviews. All interviews were translated if needed, transcribed verbatim and subsequently analyzed using Atlas.ti (see *Data analysis*).

### Recruitment participants

We enrolled individuals without any dementia-related or cognitive complaints. Participants were primarily recruited in the Rotterdam region in the Netherlands at so called “Fit Festivals” (i.e., community health events), at community centers hosting activities for healthy older adults, in the researchers’ personal and professional networks, and via snowball sampling, a recruitment technique wherein participants refer or invite other potential participants.

A purposive approach was applied within this sampling framework to enhance diversity across different characteristics. Specifically, we aimed to include participants with varying cultural backgrounds, educational levels, ages, and experiences with individuals living with dementia. Although the sample included participants from diverse backgrounds, it was not designed to exactly mirror the composition of the Dutch population in the number of participants per demographic group. The focus of inclusion was on participants of 40 years and older, as dementia prevention efforts primarily target midlife.^20^ In this stage, tackling modifiable risk factors is most effective and therefore most relevant for this study.

### Data collection

The data were collected by the authors NL and AFR through interviews that were conducted either face-to-face (n = 13), or remotely via videoconferencing (n = 7). Most interviews were conducted in Dutch (n = 15), but some were conducted in English (n = 1) and *Darija*/Moroccan Arabic (n = 4). The non-Dutch interviews were translated by the bilingual researchers. Interviews ranged in duration between 27 min to 72 min and were structured using a topic guide (see the Supplemental Material for an English translation of the topic guide). This list was developed based on literature analysis and refined after a pilot interview. Additional time was reserved for issues introduced by participants. In brief, we inquired about participants’ views on healthy aging in general, such as what that concept meant to them and what they believe is necessary to age healthily. The interviews also explored participants’ perceptions of dementia risk factors and the sources from which they had acquired their knowledge about dementia. This approach allowed for a comprehensive exploration of beliefs, experiences, and knowledge on healthy aging, dementia, and modifiable risk factors.

### Data analysis

The audio-recorded interviews were transcribed verbatim and independently analyzed by two authors (NL and AFR) using Atlas.ti. Acknowledging the potential impact of researchers’ cultural backgrounds holds significance, particularly within the context of this study where the interplay between the different cultural contexts is salient. NL was primarily socialized within Moroccan and Dutch culture, while AFR was socialized within the Somali and Dutch culture. The analysis was conducted using open, axial and selective coding as described by Babbie.^21^ In the open coding stage, researchers applied descriptive labels to portions of the transcripts to capture key concepts. During axial coding, these preliminary codes were revisited, compared, and organized into more comprehensive categories. The researchers collaboratively discussed and refined these categories. In the final selective coding stage relationships among the categories were explored to uncover recurring patterns and emerging themes.

### Participant characteristics

The sample consisted of 20 individuals from different cultural backgrounds living in the Netherlands: Moroccan (n = 6), Somali (n = 4), Surinamese Hindustani (n = 3), Turkish (n = 2), Surinamese Javanese (n = 1), Indonesian (n = 1), Iraqi (n = 1), Chinese (n = 1), and Antillean (n = 1). Demographic characteristics of the participants are displayed in Table 1. The average age of the participants was 56 years (range 40–76 years) and the sample consisted of both males (n = 12) and females (n = 8). Approximately half of the participants (n = 11) reported having a partner. Participants had varied highest completed educational levels, including participants with a primary school education (n = 5), those with high school degrees (n = 3), those who completed vocational secondary education (in Dutch “MBO4”; n = 5), and those with higher professional education (applied university, or “HBO” in Dutch; n = 7). Thirteen participants reported knowing someone with dementia in their social of familial environment.

**Table 1.** Demographic characteristics of the participants.

#	Sex	Age	Cultural background	Level of education	Knows a person with dementia
1	F	43	Turkish	Vocational secondary education	Yes
2	M	72	Surinamese Hindustani	High school	Yes
3	F	40	Moroccan *	Vocational secondary education	No
4	F	76	Surinamese Hindustani	Primary school	Yes
5	M	42	Moroccan	Vocational secondary education	No
6	F	75	Indonesian	Higher professional education	Yes
7	M	55	Turkish	Primary school	No
8	F	68	Surinamese Javanese	Vocational secondary education	No
9	F	63	Moroccan	High school	No
10	M	67	Moroccan *	Primary school	Yes
11	F	41	Surinamese Hindustani	Vocational secondary education	Yes
12	M	52	Moroccan *	Higher professional education	Yes
13	M	51	Somali	Primary school	Yes
14	M	62	Iraqi **	Higher professional education	No
15	M	49	Moroccan *	Primary school	Yes
16	M	60	Somali	Higher professional education	Yes
17	M	51	Somali	Higher professional education	Yes
18	F	56	Chinese	Higher professional education/HBO	Yes
19	M	57	Somali	Higher professional education/HBO	Yes
20	M	46	Antillean	High school	No

F: female; M: male.

*interview was conducted in Darija/Moroccan Arabic and translated by NL.

**interview was conducted in English and translated by AFR.

## Results

This section presents the findings derived from the thematic analysis. The themes are organized in a way that aligns with the I-Change Model and highlight factors influencing awareness, motivation, and action, as well as social and environmental determinants of health behavior change. The following themes will each be discussed in turn: 1) participants understanding of dementia, including awareness of risk factors and preventive strategies (awareness phase); 2) how lived experiences, such as personal health histories and encounters with illness, act as catalysts for behavior change (motivation phase); 3) structural and environmental factors that shape lifestyle choices and opportunities for prevention (contextual and action-related influences); 4) peer, familial, and cultural impacts on dementia prevention (social and cultural determinants of behavior change); 5) psychological factors that influence dementia risk perception and healthy aging (motivational and action-related determinants). Table 2 summarizes the qualitative findings structured according to the phases in the I-Change Model. The table highlights themes that emerged in the interviews related to awareness, motivation, action-related contextual influences, social determinants, and psychological factors.

**Table 2.** Overview of the study results according to the I-Change model.

I-Change phase	Emerging theme	Findings from interviews
Awareness (pre-motivational)	Understanding of dementia and risk perception	Limited awareness of modifiable risk factors.
		Emphasis on heredity as primary causes.
		Modern lifestyle as a potential risk factor.
	Biological factors	Genetic predisposition.
		Deficiencies in nutrients or malnutrition.
Motivation	Life course factors	Stress, depression, and major life events influence dementia risk.
		Experiences of trauma or health issues prompt consideration of healthier behaviors.
	Personal health experiences	Observing illness in oneself or family members as motivation for lifestyle changes.
Action – contextual factors	Lifestyle behaviors and physical environment	Healthy food choices, regular exercise, and socioeconomic conditions support healthy aging.
		Smoking, alcohol use, physical impairment, and need for relaxation possibly relevant for some individuals.
	Cognitive activity	Learning new skills is beneficial.
		Sustaining cognitive activity through daily tasks is important.
Social – cultural determinants	Social interactions or activities	Engagement with family, friends, and community, as well as social accountability, are important influences on health behaviors.
	Cultural norms and values	Cultural expectations and religious practices shape health behaviors.
Psychological factors (motivation and action related)	Mindset, personality, and coping	Personal responsibility, mindset, resilience, and personality traits are important determinants of health-promoting behaviors.
		Stress management and self-regulation may also be relevant.

### Emerging themes from the thematic analysis

#### Theme 1: Dementia Awareness (pre-motivational phase)

Participant 12 reported that they were unaware of modifiable dementia risk factors. This reflects an initial low dementia literacy in the pre-motivational phase as described in the I-Change Model:

‘To be honest I’m very unfamiliar with it. I could make up a good story, but that's not what you are looking for.’ (Participant 12)

Other participants described dementia primarily as a condition caused by biological vulnerability rather than one that could be prevented, emphasizing heredity and genetic predisposition over lifestyle or modifiable risk factors:

‘I once read somewhere that it's in your genes. You’re kind of a carrier of those genes. Everyone has them, but some people may be more predisposed than others.’ (Participant 13)

‘I think it's hereditary. Just bad luck really — like a sudden mutation in your genes.’ (Participant 14)

‘Well, I don’t think it really has a cause. It's something natural, and as far as I know, it's also hereditary.’ (Participant 10)

These participants framed dementia through a biological lens, suggesting that genetics dominates over modifiable factors. Participant 13 stated a sense of inevitability by phrasing it as “*you are kind of a carrier of those genes”* and therefore suggesting limited room for modifiable risk factors to influence disease onset. Participants 16 and 19 introduced physiological explanations, such as deficiencies in nutrition. This may introduce a greater openness to modifiable risk factors:

‘I suspect it has to do with a deficiency of certain substances in the body. If there's a shortage of specific substances, there's a good chance it could cause damage in certain parts of the body, such as the brain.’ (Participant 16)

‘A risk factor that hasn’t been taken into account is malnutrition at a young age. (…) That this can delay the development of your brain. Or the malnutrition can slow down the growth potential of your brain.’ (Participant 19)

By framing dementia as stemming from “*a deficiency in the body”*, this perspective implies a potentially more open attitude toward prevention efforts. While participant 16 did not elaborate on what these deficiencies entailed, participant 19 later in the interview mentioned a calorie deficit and vitamin deficiencies as possible contributors to the development of dementia.

After reflecting on their own perceptions of dementia risk, participants were presented with an overview of risk factors^3^ including high blood pressure, smoking, and social isolation. Many participants were surprised by some of these factors, acknowledging that they had not previously recognized their association with dementia. This indicates a possible gap in dementia awareness/-literacy and highlighting an opportunity for awareness-building interventions:

‘Yes, that being overweight or having few social contacts increases your risk of getting dementia — that really surprised me. (…) If your body is already used to all those things, it can also be harder to readjust when you want to age healthily.’ (Participant 3)

One participant challenged limited social contact as a risk factor by emphasizing that the quality of the relationships and social interactions may be more important than their quantity:

‘You don’t need to have a very active social life to slow down decline, I think. It also depends on who you spend time with, right? I mean, if you have 30 people who hang out at the coffeeshop [cannabis retail store in the Netherlands] every day, and you’re there too even though you don’t smoke, I don’t think those are the right social contacts. So, it's better not to have those and instead keep stimulating your brain in other ways. I don’t necessarily think having a larger social network is important. It really depends on your attitude.’ (Participant 11)

This participant also highlighted the importance of individual agency and mindset by stating “*it really depends on your attitude”*. This suggests a personal motivation to maintain a healthy brain, which for this participant may be just as important as social connections.

These findings together demonstrate that the pre-motivational phase is strongly influenced by existing knowledge, beliefs and dementia literacy. This in turn shapes whether individuals perceive dementia as preventable.

#### Theme 2: Lived health experiences as drivers of health behavior change (motivational phase)

Many participants expressed limited awareness of specific modifiable risk factors for dementia but elaborated that (personal) experiences with declining health served as a medium for adopting healthier behavior. This is in line with the motivational phase of the I-Change model. Notably, these health behavior changes were seldom framed in direct relation to dementia prevention but were instead linked to other health concerns such as cardiovascular health or general wellbeing:

‘I am Hindustani. I don’t know if you’re familiar with all the chronic illnesses we can have? High blood pressure, kidney issues, all sorts of things. Well, I’m a product of that too—things I might be more prone to because I’m Hindustani. So, if I neglect all of that, it will all deteriorate more quickly, and I’ll be more likely to, for example, develop heart problems or something like that.’ (Participant 11)

Participant 2 highlighted that personal effort and responsibility are central motivational factors in managing health:

‘You get so much information, and if you still continue with an unhealthy lifestyle, then I just think that's stupid. At least try. And if it doesn’t quite work out, then it doesn’t – but you should still make the effort to try.’ (Participant 2)

Another participant highlighted that lasting change requires a more fundamental shift:

‘Uhm to stick with it, I think you actually have to change your way of thinking. I think you need to change your mindset in order to really be able to keep it up. (…) The way you think about things can determine whether something goes well or not.’ (Participant 3)

The examples highlight how cues to action contribute to the formation of intentions to adopt healthier behaviors. The participant's reflections suggest that health behavior change is not only influenced by knowledge or information provision, but also intertwined with personal beliefs, experiences, and mindset. Dementia may lack the immediacy required to take action unless tied to more concrete health experiences. Some participants connected the lived experiences of others to changes in their own behaviors or mindset. Experiences of others also acted as powerful cues to action:

‘Exactly, when I see someone my age who has problems with obesity, diabetes, or cholesterol issues because of unhealthy eating, it motivates me to start living healthily. (…) Then you want to make the change for yourself.’ (Participant 13)

‘Well, of course, I see enough people around my age and even younger. Who have become socially isolated, who live unhealthily, look unhealthy, get sick, suffer from all kinds of illnesses, diabetes, cholesterol, you name it. Yes, so that's what I fear. If you don’t take care of your body and your mind, then I’m afraid things will go really wrong.’ (Participant 17)

Another respondent shared a reflection on the health struggles of a close family member as a direct cue to behavior change. This further illustrates the influence of personal cues on action within the I-Change framework:

‘When a close family member of mine became ill and had a heart attack. It was really a wake-up call for me. He had a very unhealthy lifestyle and was increasingly struggling with his breathing because of that. Since seeing that, I’ve been paying more attention to my diet and exercise.’ (Participant 9)

These findings highlight the dynamic interplay between illness experiences, perceived risk, and motivation. It shows how dementia literacy alone may not be sufficient to drive preventive behavior. Personal experiences and observations of others can serve as important triggers and moves individuals from awareness to intention and ultimately to action, consistent with the phases of the I-Change Model.

#### Theme 3: Social and structural determinants of health (action phase)

A recurring theme in the interviews was the idea that leading a healthy lifestyle – in the context of dementia risk reduction and prevention – goes beyond having the right knowledge. Several participants expressed that certain people are better positioned to invest in their health due to social determinants of health. This aligns with the I-Change Model's recognition that behavior is influenced not only by awareness but also by environmental and contextual factors. Participants elaborated on socioeconomic status, accessibility of healthy food, and physical environment as facilitators or barriers. One participant illustrated this with the example of a public figure:

‘Cristiano Ronaldo, for example. You can see that his financial freedom allows him to fully focus on his work. It also means he can afford healthy food. That's why he looks the way he does at his age. He has the means to invest in himself.’ (Participant 5)

This perspective was reinforced by another participant who pointed to the difficulty of sustaining a healthy diet on limited economic resources:

‘Fortunately, I don’t have to worry about what it costs to stick to my diet. But I can imagine that if you’re poor it is definitely more difficult to do so.’ (Participant 11)

Social determinants of health also emerged in relation to cultural and geographic context, which shape how people live and make health-related choices. For example, participant 5 compared Morocco and the Netherlands, noting that the traditional lifestyle in Morocco was perceived as healthier:

‘People in Morocco have a much healthier lifestyle than here. They don’t have money for fast food and they don’t eat out every day. Maybe their misfortune is a blessing in disguise, because they grow their own food. They eat a lot of organic things.’ (Participant 5)

For participant 6, healthy aging was also tied to psychosocial factors, such as breaks from their regular routine:

‘That's why it's important to schedule vacations regularly. If you don’t do that and don’t get away from your environment, it's hard to build resistance to keep going and have good health later in life.’ (Participant 6)

In addition to financial and environmental conditions, (dis)ability was also identified as an important factor shaping healthy aging, including impairments in vision or mobility:

‘I think if you’re disabled – if you have visual problems or physical issues that make you dependent – it becomes more difficult. If your mobility or vision is limited, everything gets harder.’ (Participant 11)

These insights show how individuals situate healthy aging within a broader social and personal context. Awareness and motivation alone are insufficient to practice preventive behaviors. Contextual determinants need to be considered alongside dementia awareness/literacy when understanding preventive behavior.

#### Theme 4: Peer, familial, and cultural impacts on healthy aging

In addition to exploring participants’ beliefs about the causes of dementia and conditions of healthy aging, we also inquired about the role of social relationships in shaping health-promoting behaviors. This is in line with the I-Change model's recognition that social influences shape intention formation and behavior execution. Participants reflected on how healthy aging is tied to social interactions and how peer connections can serve as an external accountability mechanism. The participants described how friends, family, and cultural norms could encourage (or discourage) them to sustain a healthy lifestyle over time. For example:

‘I’ve noticed that I’m someone who really needs social control to do well. Otherwise, I’d be far too easy on myself. If no one checks on me, I’ll just stay on the couch. But if someone calls and persuades me [to go and exercise], it starts to feel more like an obligation.’ (Participant 3)

For this participant, engaging in health-promoting behaviors was more feasible if they experienced a shared sense of commitment (in line with the motivational cue in the I-Change model). Shared expectations between individuals were described as a way of reinforcing lifestyle practices. Another participant highlighted the importance of regular discussions with friends to serve as emotional support and keep a balanced outlook on health-promoting behaviors:

‘I think the most important thing is to keep talking with your friends. It's not like I always do everything perfectly, but it's about what your automatic reaction is – your natural way of acting. That unhealthy behaviors are the exception, not the rule. We are all human, and I can enjoy the less healthy things too, but I feel better when I feel good in my own skin. That's what healthy living does for me, and I try to share that with my friends.’ (Participant 5)

In contrast, another participant noted that striving to maintain a healthy lifestyle could bring up feelings of “amazement” or provoke comments from others within the social network. Reflecting on her own experience, participant 8 shared the following:

‘At yoga class I would always be the oldest person attending. They would keep on saying: ‘It's so good that you’re doing this at your age!’. When you take good care of your body it is very possible at my age.’ (Participant 8)

This demonstrates a strong sense of personal agency and selfcare in relation to healthy aging. Her approach may also serve as a source of inspiration for others (i.e., people in her yoga class) and challenge societal expectations or views about older age.

Additionally, cultural expectations and fear of judgement can act as barriers to health-promoting behaviors, particularly when such actions are perceived as deviating from “group norms”. It underscores the importance of considering socio-cultural context, including the potential restrictive group norms, in the design of interventions to support healthy aging. A community can play a dual role. While belonging can offer encouragement and a sense of identity, it can also enforce conformity through peer pressure. These accounts illustrate how community expectations shape what behaviors are seen as acceptable, which in turn influences how people understand their own possibilities of healthy aging. Participant 5 reflected as follows:

‘I think it's simply a combination of your lifestyle and the supportive environment you have. You might have the motivation, but that still makes it very difficult. Suppose being overweight is the norm in your environment. Then you might know it's not healthy, but if you follow a different lifestyle, that could also create a certain image of you in an environment where that's not the norm.’ (Participant 5)

The challenge participants described was not only about personal effort, but also about navigating social expectations and about negotiating one's identity within a social context that may not reward healthy behaviors. It is about the subtle cost of deviating from group norms. This reflection shifts the discussion from internal motivation for healthy aging to external pressure to conform to what is seen as “normal”. This shows that healthy aging is not only a matter of personal discipline or access to resources, but also of navigating how social identity and cultural belonging shape both the feasibility and the potential social costs of health-promoting behaviors.

#### Theme 5: Psychological factors in relation to dementia risk (motivation and action)

Participant 5 and 12 touched upon psychological factors, such as personality, intentions, discipline, and stress, in discussing dementia risk. These factors reflect both motivational influences (intention formation) and action-related skills (self-regulation and persistence):

‘I have the feeling — it's just a thought — but I think it has to do with the way we live. More stress, more rush, and we want so much. At a certain point, the upstairs [mind] just can’t handle it anymore, I think.’ (Participant 12)

‘It's about personality. One person may have a strong personality, be proactive, and unafraid to try new things, even if it means learning through trial and error. Someone else might be more reserved in their actions, which also has its own impact.’ (Participant 5)

When reflecting on possible risk factors for dementia, participant 5, 8 and 19 suggested other potential influences of diseases such as trauma, stress, and lack of sleep:

‘One possible risk factor could be the traumas experienced at a young age.’ (Participant 19)

‘What I especially miss in this list is stress. It's striking that it isn’t mentioned. It's the cause of almost everything.’ (Participant 8)

‘I’m actually surprised that sleep isn’t included [in the list of risk factors]. (…) For me, staying up at night and sleeping in until late doesn't belong to a healthy lifestyle and aging. You can also see that God created us in such a way that our natural rhythm only allows us to sleep at night, not during the day. When that rhythm is disrupted, you notice it in your health.’ (Participant 5)

Closely tied to these perceptions is the importance of psychological resilience and boundary setting as protective factors. One participant emphasized the importance of rest and managing demands to prevent (mental) overload:

‘I think it's really just about taking your rest and not doing too much at once. That you think…sometimes you just have to say, ‘Okay guys…’ – you really have to set a boundary for yourself, like, ‘This is just as much as I can handle.’ Not too much, but also not saying yes to everything just out of loyalty. At some point, you’ll hit a wall and realize maybe it was all just a bit too much. You learn from trial and error. And eventually, you reflect on it and take a step back.’ (Participant 15)

Taken together, these reflections underscore the broader context of psychosocial factors and personality in understanding health- and dementia risk factors. Awareness of risk alone may be insufficient if individuals lack coping strategies, resilience, or self-regulatory skills. Conversely, psychological well-being and self-management may enhance motivation and facilitate the adoption of health-promoting behaviors, including those relevant to dementia prevention.

## Discussion

This study explored individuals’ knowledge of dementia, their understanding of, and beliefs about modifiable risk factors. The five main themes that emerged from the qualitative analysis are lay narratives of dementia and perceived risk factors (awareness in the I-Change model); lived experiences as drivers of dementia-related health behavior change (motivational cues to action); the influence of social determinants of health on dementia prevention efforts (action phase in the I-Change model); peer, familial, and cultural impacts on healthy aging (social – cultural determinants); and the perceived role of psychological influences on dementia risk and healthy aging (psychological factors).

One of the main findings was that participants were either unable to name any risk factor for dementia or believed that whether someone develops dementia is determined mainly by their biological vulnerability/genes, rather than believing it to be a condition that can be prevented (awareness I-Change phase). This finding in a diverse population in Europe echoes previous findings among diverse populations in the U.S. For example, Tran et al.^22^ found that, the majority of 216 Latino Americans was uncertain about the exact cause of the Alzheimer's disease and genetics was the most frequently endorsed cause—although several participants also highlighted the multifactorial nature of Alzeheimer′s disease. Similarly, in Black populations in the U.S., genetics is often endorsed as the (main) cause of Alzheimer′s disease.^23^ While international comparisons are informative, this study extends the evidence base by showing how similar explanatory models are present among culturally diverse groups in the Netherlands. Notably, some participants suggested risk factors not currently endorsed by the scientific community or questioned aspects of established risk factors.

Sleep disturbance and stress were two “novel” factors participants considered important for dementia risk in the awareness phase. Both factors are increasingly discussed as potential modifiable risk factors for dementia but have not been endorsed definitively by the scientific community due to a lack of consistent evidence.^1^ Regarding sleep, debate is ongoing about a potential reverse causation bias (poor brain health resulting in poorer sleep as opposed to vice versa). Research into (post-traumatic) stress as a risk factor for dementia is emerging but not conclusive.^24^ The finding that participants identified these factors illustrate how personal- and lived experiences inform their explanatory models (motivation phase in the I-Change model), even where biomedical consensus remain unsettled. Such beliefs may nonetheless provide entry points for culturally tailored discussion about dementia prevention, particularly if communication resonates with participants’ explanatory models of dementia, which may differ across cultures.^13^ Incorporating these culturally and personally relevant models into awareness strategies may increase their perceived relevance and help make preventive messages resonate with the target population. Social interactions were considered to potentially have both positive and negative effects on healthy behaviors; participants described the benefits from a shared sense of commitment to health behaviors, while also describing barriers in defying social or group norms. For example, promoting social interactions to combat stress or suggesting (psychological) support in processing past traumatic experiences may result in reduced rates of social isolation and depression– both established dementia risk factors.

Some participants highlighted social inequalities, such as economic disadvantage, as barriers to healthy aging. These factors align with the key drivers of health disparities outlined in the recent NIMHD Research Framework^19^ and are particularly relevant when considering risk for Alzheimer's disease. Socioeconomic status, for instance, shapes early life conditions that can have lasting effects on health trajectories, influencing both risks and opportunities across the life course.^19^ In the case of dementia, intergenerational influences (e.g., growing up in less health-promoting environments) may impact the development of health-related behaviors, including engagement with preventive care and access to health-promoting resources, factors that are important in reducing risk (social – cultural determinants phase in the I-Change model). Health inequalities may also arise from socialization processes through which individuals adopt norms, values, and behaviors shaped by peers and broader societal influences.^25^ Moreover, social inequalities, and the resulting inequities, can have lasting effects across the lifespan, negatively influencing brain health and increasing both the risk and disparities related to Alzheimer's disease.^26^

In the interviews, it became clear that participants’ behaviors were often guided by experiences of poor health, either in their personal lives or in the lives of others. Such illness behavior*,* in which action is triggered by symptoms, as opposed to health behavior, in which actions are undertaken to sustain good health, may have important consequences for dementia prevention policy or strategies. Participants’ narratives often emphasized reactive rather than proactive views of health. Awareness efforts may therefore benefit from acknowledging illness-driven accounts as a starting point and linking them carefully to preventive messages. Additionally, if individuals attribute the development of dementia primarily to genetic factors, they may perceive the condition as inevitable and beyond personal control.^27^ Such explanatory models could diminish receptivity to prevention information, under the assumption that available actions are unlikely to alter genetic risk. Communication strategies should therefore highlight that dementia is a multifactorial condition and that risk can be modified, without dismissing lived experiences.

This study has several strengths. First, a highly diverse sample was recruited in terms of culture, education, and age, reflecting the diversity within the Dutch society. An effort was made to include participants with limited Dutch proficiency who are often underrepresented in research. Second, the rich narratives in this study provided a more in-depth exploration of the ways in which participants understood their dementia risk which helps to supplement findings from existing (global) quantitative research. The large diversity among the participants may also have carried a risk, as it cannot be ruled out that some culture-specific factors may have been missed which may not have been endorsed by the few participants from that specific cultural group that were interviewed. Furthermore, as is the case with most qualitative research, the researchers’ own position may have influenced the study in subtle ways. The two interviewers had both shared and unique cultural backgrounds and interviewed participants with both similar and different cultural backgrounds. The cultural match between the interviewer and interviewee can subtly shape the way or the degree to which participants speak about specific health beliefs or behaviors, both positively (e.g., resulting in participants being more open) and negatively (e.g., participants not sharing specific thoughts out of shame).

In conclusion, this study highlights the complex interplay between individual beliefs, (cultural) narratives, and social aspects in shaping how people perceive dementia and the role of preventive strategies. While awareness of dementia is growing, many individuals may lack knowledge about specific risk factors and continue to view dementia primarily as a genetically determined condition. Such perceptions may limit receptivity to prevention messages. However, participants’ narratives also reveal important opportunities for public health. These include incorporating lived experiences, acknowledging illness narratives, and aligning messages with culturally embedded beliefs about health. To effectively reduce dementia risk at a population level, especially among underserved groups, awareness campaigns and communication strategies should be context-sensitive, socially responsive, and resonate with diverse explanatory models.

## Supplemental Material

sj-pdf-1-alz-10.1177_13872877261443983 - Supplemental material for Beliefs, barriers, and behaviors: Exploring dementia prevention perspectives in culturally diverse populations in The Netherlands Supplemental material, sj-pdf-1-alz-10.1177_13872877261443983 for Beliefs, barriers, and behaviors: Exploring dementia prevention perspectives in culturally diverse populations in The Netherlands by Najoua Lazaar, Awaale F. Rirash, Janne M. Papma, Francesco U. S. Mattace Raso and Sanne Franzen in Journal of Alzheimer's Disease
