# Supplementary material for: Beliefs, barriers, and behaviors: Exploring dementia prevention perspectives in culturally diverse populations in The Netherlands
Source: J Alzheimers Dis. 2026 Apr 23;111(4):1725–34. doi: 10.1177/13872877261443983 (PMC13234305; doi:10.1177/13872877261443983)
Supplement: sj-pdf-1-alz-10.1177_13872877261443983 - Supplemental material for Beliefs, barriers, and behaviors: Exploring dementia prevention perspectives in culturally diverse populations in The Netherlands [file sj-pdf-1-alz-10.1177_13872877261443983.pdf]

Topic guide: healthy lifestyle and dementia (English translation)

General information

- ☐ The interviewer introduces themselves.
- ☐ Practical information about the interview.
- ☐ Answer any preliminary questions.
- ☐ The purpose of the interview is explained.
- ☐ Obtain informed consent.

Introduction

The population of older adults in the Netherlands/Europe is increasing. With this aging, the vulnerability to develop health problems also increases. This includes a higher risk of diseases such as dementia, cancer, and cardiovascular conditions. The aim of this study it to gain more insight into people's perspectives on healthy aging. We are also interested in the role of culture and family, given that little research has been conducted among people with a migration background.

General questions on healthy aging

- What comes to mind when you think of 'healthy living'?
  - And what about 'unhealthy living'?
- Are there changes you would like to make to live more healthily?
  - Why would you like to make these changes?
- Have you ever made changes in the past to live more healthily?
  - How do you reflect on that experience?
- Do you actively think about healthy aging?
  - What do you do to grow older in good health?
- What could happen if you did not pay attention to healthy aging?

Knowledge about dementia

- Could you tell me what you already know about dementia?
- How did you first find out what dementia is?
- In your opinion, what could be a cause of dementia?
  - [Space to elaborate on explanatory models of dementia.]
- Do you think it is possible to reduce dementia risk ?
  - Yes: please explain (how)?
  - No: could you explain why (probably) not?
- If we turn the question around: What do you think contributes to the risk of dementia?
- I would like to tell you several established risk factors associated with dementia. What strikes you about this list, and why?
  - Show the 10 risk factors as illustrated by Alzheimer Nederland (see infosheet below or Risicofactoren dementie | Alzheimer Nederland).
  - Do you think there are any dementia risk factors missing from this list?

Social influence

- Could you tell me more about your social life?
  - For example, are you involved in groups or activities such as a (sports) club, mosque/church, or do you do volunteer work?
- Let's suppose you wanted to make changes in order to age more healthily. Where would you start?
  - What would you need for this?
  - What would help you to maintain it?
  - If not already mentioned: what role do your family, friends, or acquaintances play in this?

- Suppose you wanted to make changes to age more healthily. How would you feel about doing this entirely on your own?
- Are there things that your family, friends, or people you admire (perhaps unconsciously) do to age healthily? If so, would you adopt these practices yourself?
- Are there things you would recommend to your family, friends, or acquaintances to promote healthy aging?
- Does your family have an influence your motivation to age healthily?
    - In what way do they demonstrate this?
    - Does dementia prevention play a role here?
- Is healthy living stimulated within your culture?
    - In what way?
- Does religion or spirituality play a role in healthy aging for you? Do you think it plays a role for others?

Thank you for your answers. Finally, we have a scenario that we would like to discuss with you:

Imagine a friend wants to age more healthily by exercising more and eating better.

- What would they need, in your opinion/view, to accomplish this and to maintain it?
    - What role could their family or friends play in supporting them? / Would you personally do something to help them?
- What challenges do you think they might encounter if they want to exercise more and eat more healthily?
- Do you think it is more difficult for some people to age healthily than for others?
    - For example, people with certain characteristics or circumstances?
    - Who comes to mind, and why?

Demographic characteristics of the interviewee

- ☐ Gender
- ☐ Age
- ☐ City of residence
- ☐ Do you have a partner?
    - If yes, do you live together or are you married?
- ☐ Do you consider yourself religious?
- ☐ To which cultural group do you belong?
- ☐ Where were your (grand)parents born?
- ☐ What is the highest level of education you have completed?
- ☐ Do you know people with dementia in your immediate environment (family, friends, others you are in contact with)?

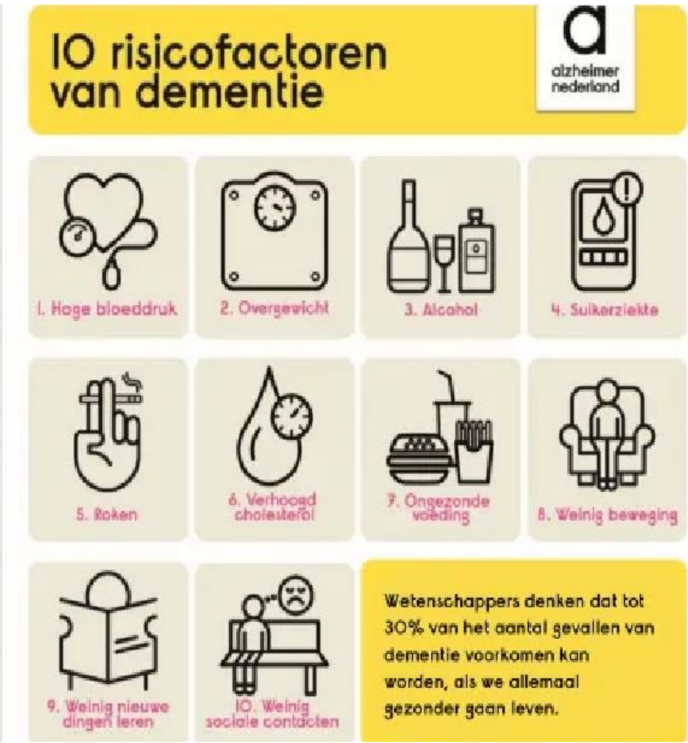
10 risicofactoren van dementie
alzheimer nederland
1. Hoge bloeddruk
2. Overgewicht
3. Alcohol
4. Suikerziekte
5. Roken
6. Verhoogd cholesterol
7. Ongezonde voeding
8. Weinig beweging
9. Weinig nieuwe dingen leren
10. Weinig sociale contacten
Wetenschappers denken dat tot 30% van het aantal gevallen van dementie voorkomen kan worden, als we allemaal gezonder gaan leven.
